# Hybrid Explainable Artificial Intelligence Models for Targeted Metabolomics Analysis of Diabetic Retinopathy

**DOI:** 10.3390/diagnostics14131364

**Published:** 2024-06-27

**Authors:** Fatma Hilal Yagin, Cemil Colak, Abdulmohsen Algarni, Yasin Gormez, Emek Guldogan, Luca Paolo Ardigò

**Affiliations:** 1 Department of Biostatistics and Medical Informatics, Faculty of Medicine, Inonu University, Malatya 44280, Turkey; cemil.colak@inonu.edu.tr (C.C.); emek.guldogan@inonu.edu.tr (E.G.); 2 Department of Computer Science, King Khalid University, Abha 61421, Saudi Arabia; a.algarni@kku.edu.sa; 3 Department of Management Information Systems, Faculty of Economics and Administrative Sciences, Sivas Cumhuriyet University, Sivas 58140, Turkey; yasingormez@cumhuriyet.edu.tr; 4 Department of Teacher Education, NLA University College, 0166 Oslo, Norway

**Keywords:** diabetic retinopathy, targeted metabolomics, hybrid explainable artificial intelligence, explainable deep learning, biomarkers

## Abstract

Background: Diabetic retinopathy (DR) is a prevalent microvascular complication of diabetes mellitus, and early detection is crucial for effective management. Metabolomics profiling has emerged as a promising approach for identifying potential biomarkers associated with DR progression. This study aimed to develop a hybrid explainable artificial intelligence (XAI) model for targeted metabolomics analysis of patients with DR, utilizing a focused approach to identify specific metabolites exhibiting varying concentrations among individuals without DR (NDR), those with non-proliferative DR (NPDR), and individuals with proliferative DR (PDR) who have type 2 diabetes mellitus (T2DM). Methods: A total of 317 T2DM patients, including 143 NDR, 123 NPDR, and 51 PDR cases, were included in the study. Serum samples underwent targeted metabolomics analysis using liquid chromatography and mass spectrometry. Several machine learning models, including Support Vector Machines (SVC), Random Forest (RF), Decision Tree (DT), Logistic Regression (LR), and Multilayer Perceptrons (MLP), were implemented as solo models and in a two-stage ensemble hybrid approach. The models were trained and validated using 10-fold cross-validation. SHapley Additive exPlanations (SHAP) were employed to interpret the contributions of each feature to the model predictions. Statistical analyses were conducted using the Shapiro–Wilk test for normality, the Kruskal–Wallis H test for group differences, and the Mann–Whitney U test with Bonferroni correction for post-hoc comparisons. Results: The hybrid SVC + MLP model achieved the highest performance, with an accuracy of 89.58%, a precision of 87.18%, an F1-score of 88.20%, and an F-beta score of 87.55%. SHAP analysis revealed that glucose, glycine, and age were consistently important features across all DR classes, while creatinine and various phosphatidylcholines exhibited higher importance in the PDR class, suggesting their potential as biomarkers for severe DR. Conclusion: The hybrid XAI models, particularly the SVC + MLP ensemble, demonstrated superior performance in predicting DR progression compared to solo models. The application of SHAP facilitates the interpretation of feature importance, providing valuable insights into the metabolic and physiological markers associated with different stages of DR. These findings highlight the potential of hybrid XAI models combined with explainable techniques for early detection, targeted interventions, and personalized treatment strategies in DR management.

## 1. Introduction

Diabetic retinopathy (DR) is a common microvascular complication of diabetes mellitus (DM) and a leading cause of vision loss in diabetic patients [1]. DR is associated with multiple risk factors, including hyperglycemia, hyperlipidemia, hypertension, and genetic factors [2]. DR can be classified into two distinct stages: non-proliferative DR (NPDR) and proliferative DR (PDR), based on the presence or absence of neovascularization [3]. A major complication of diabetes is DR, which damages the retina and can cause blindness. Leakage, hemorrhage, and irregular vessel development are all symptoms that can be caused by high blood sugar levels, which can also alter the blood vessels in the retina. Early detection is essential for efficient management of disaster recovery, which continues through the stages. It is possible to avoid DR or reduce its growth by maintaining the effective management of diabetes through diet, exercise, and medication. It may be necessary to undergo laser therapy or surgery in more severe situations to safeguard one’s eyesight. To prevent diabetes patients from experiencing vision loss, it is necessary to participate in early diagnosis and vigorous care [4]. Epidemiological studies have identified several risk factors associated with the development and progression of DR, including a higher body mass index, a higher waist-to-hip ratio, smoking, congestive heart failure, chronic renal disease, hypertension, and poor glycemic control. The prevalence of DR varies depending on the population and type of diabetes, with rates ranging from 5.67% in prediabetes to 41.1% in diabetes patients at tertiary care centers. Risk factors implicated across various populations and diabetes types include obesity, hypertension, longer diabetes duration, insulin therapy, neuropathy, nephropathy, and dyslipidemia [5,6].

Current DR treatment strategies focus on preventing its progression and managing complications to preserve vision. Glycemic control is key, as maintaining optimal blood glucose levels can significantly reduce the risk of developing DR and slow its progression. Anti-VEGF (Vascular Endothelial Growth Factor) therapy with repeated doses of drugs such as ranibizumab and aflibcept can reduce diabetic macular edema by reducing inflammation and preventing abnormal blood vessel growth used to treat edema (DME) and proliferative diabetic retinopathy (PDR). Laser photocoagulation, which masks leaky blood vessels and reduces the risk of severe loss, remains the mainstay of therapy, especially for PDR and focal DME. Intravitreal steroids, such as triamcinolone acetonide, are especially useful and anti-VEGF is used to reduce inflammation in non-clinical cases. In advanced PDR, vitrectomy surgery may be required to eliminate vitreous hemorrhage or tractional retinal detachment and restore normal retinal anatomy and function. Several advanced methods are available for detecting and monitoring retinal changes in the diagnosis of DR. Ophthalmology using direct and indirect techniques allows physicians to visualize the retina and detect symptoms such as microaneurysms, bleeding, and exudation. Fundus imaging provides detailed, useful visualization of the retina for recording and tracking DR progress, and is commonly used in assessment programs [7,8].

Metabolomics profiling, which involves the comprehensive quantitative analysis of small-molecule metabolites in biological specimens like blood and urine, has advanced significantly in recent years [9]. The metabolic phenotype reflects the intricate interplay between genetic and environmental factors and provides valuable insights into the pathophysiological conditions of various diseases, including DR [10]. Several large-scale metabolomics profiling studies have been conducted to identify metabolites associated with disease progression, particularly in DR. However, there is still a need to identify additional metabolites that could serve as reliable biomarkers for DR progression and aid in the early treatment and prevention of diabetic complications [11]. Additionally, targeted metabolomics was employed to analyze the metabolome data of DR patients, revealing significant differences in the concentrations of specific metabolites among non-DR, NPDR, and PDR type 2 DM (T2DM) patients. This approach provides valuable insights into the metabolic changes associated with different stages of DR in diabetic individuals [12].

Hybrid explainable artificial intelligence (XAI) refers to the merging of several AI methodologies or models to boost performance. Hybridity is more prominent in AI research because of the various needs of the scientific, public, and commercial sectors. A study focused on mask-wearing status employed a mixture of convolutional neural networks, including SqueezeNet, InceptionV3, VGG16, and VGG19, together with several machine learning (ML) models, to develop hybrid models for classification, achieving excellent accuracy [13].

In the present study, we propose a methodology that integrates a hybrid AI model and XAI approaches for early diagnosis and determination of metabolomic biomarkers of DR in patients with T2DM. Unlike existing methods that usually rely solely on clinical parameters or traditional imaging techniques, our approach integrates advanced machine learning algorithms to identify specific metabolites with varying concentrations among individuals with NDR, NPDR, and PDR. This research attempts to uncover distinct biochemical signatures related to different subclasses of DR, providing important molecular-level insights into disease etiology and development. Our study integrates various learning algorithms with a hybrid innovative approach to increase prediction accuracy and sensitivity, and this methodology provides a more robust and reliable tool for clinical decision-making in DR. This innovative methodology may enable the establishment of tailored treatment strategies and more successful screening procedures for DR.

## 2. Material and Methods

### 2.1. Study Participants and Selection Criteria

This study conducted a cross-sectional analysis of a cohort of 317 individuals diagnosed with T2DM. The objective was to investigate the blood biomarkers that may be linked to DR. The research group was divided into several groups based on the severity of DR. It included 143 patients with NDR, 123 patients with NPDR, and 51 patients with PDR. The diagnoses were carefully verified by thorough dilated fundus examinations performed by an expert in retinal diseases, guaranteeing the accuracy of the classifications for diabetic retinopathy. The reliability of the results can be considered high due to the thoroughness of the dilated fundus examinations, the expertise of the retinal specialist, and the systematic approach to obtaining and preserving serum samples at −80 °C. Systematically, serum samples were obtained from all participants, including individuals diagnosed with DR and those without, to examine possible disparities in biomarkers that may be associated with the advancement of DR. The samples were promptly kept at a temperature of −80 °C to maintain their integrity, following the most rigorous standards of biological sample preservation. The investigation was done with rigorous adherence to ethical norms [14]. MetSizeR was used to determine the necessary sample size for this investigation using the PPCA model and a false discovery rate of 0.05. With a minimum of 14 patients in each group, an estimated total sample size of at least 42 patients was obtained. The sample size was higher than that predicted by MetSizeR [15], a method used to estimate sample size in metabolomics investigations, despite the challenge of finding patients who fulfilled the study’s inclusion criteria.

Inclusion Criteria
T2DM Diagnosis: To participate, participants had to have a verified T2DM diagnosis.
Participants must be adults between the ages of 18 and 75.
Classification of Diabetic Retinopathy: Participants were classified using comprehensive dilated fundus exams into one of the following groups: Diabetic Retinopathy Absent; Not Proliferative Diabetic Eye Disease; and Growth-oriented Diabetic Eye Disease. For a participant to take part in the research, written informed consent was required.
Exclusion Criteria
Individuals suffering from other retinal conditions, such as age-related macular degeneration or retinal vein occlusion, were not allowed to participate in this study.
Severe Systemic Illness: Research participants who may have a significant impact on the study’s results, such as cancer or severe cardiovascular problems, were disqualified.
History of Ocular Surgery: Those who have had eye surgery performed within the last year (apart from cataract surgery) were not allowed to participate.
Pregnancy: Because pregnancy may have confounding effects on metabolic profiles, women who were pregnant were not allowed to participate.
Incapacity to Give Informed Consent: Individuals who, for whatever reason, were incapable of giving informed consent because of cognitive impairments were not allowed to participate.

### 2.2. Metabolomics Profiling

Serum samples from T2DM patients underwent analysis using a targeted metabolomics approach. Liquid chromatography (LC) and flow-injection analysis (FIA)–mass spectrometry (MS) were conducted using the AbsoluteIDQ1 p180 Kit from BIOCRATES Life Sciences AG, Innsbruck, Austria. Quality control measures were implemented to select metabolites for further analysis based on significant differences in their concentrations among the different patient groups. The serum samples were analyzed using the API 4000 QTRAP LC/MS/MS system (Applied Biosystems, Foster City, CA, USA) and the Agilent 1200 HPLC system (Agilent Technologies, Santa Clara, CA, USA) following standard protocols. Calibration standards and quality controls were established using the AbsoluteIDQ1 p180 Kit, and data quality checks were performed to ensure accurate metabolite concentrations. A total of 122 metabolites meeting the quality control criteria were selected for subsequent statistical analyses [14].

### 2.3. Stages of an Explainable Deep Learning Model

#### 2.3.1. Data Collection and Preparation

In this study, the T2DM dataset, which has 317 samples from three different classes, was used to train and test ML models. Upon examining the dataset, it was noted that there were some missing values. The missing values in the dataset were addressed by calculating the average of the complete data within the relevant column [16].

#### 2.3.2. Model Selection

**Figure 1:**
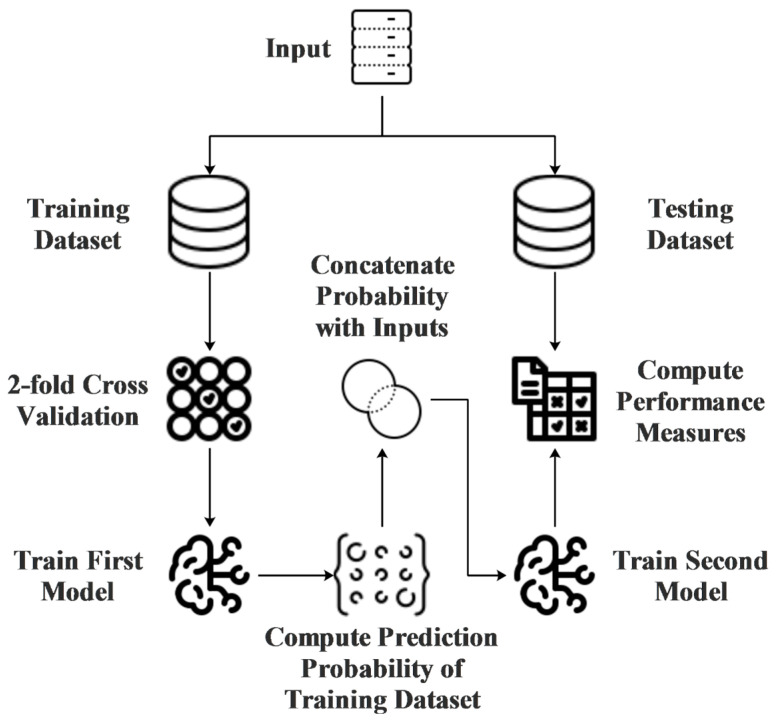
This figure illustrates the architecture of the proposed two-stage ensemble hybrid model used in the study [The first stage involves training a Support Vector Classifier (SVC) on the dataset using 2-fold cross-validation to compute prediction probabilities. These probabilities are then used as additional features for the second stage, where several other machine learning models (Multilayer Perceptron (MLP), Random Forest (RF), Decision Tree (DT), and Logistic Regression (LR)) are trained. This two-stage process aims to enhance prediction accuracy by leveraging the strengths of multiple learning algorithms].

A neural network architecture suitable for structured clinical data, such as multilayer perceptrons (MLP), was chosen for the targeted task. These models were capable of efficiently handling high-dimensional data and complex relationships between features. In this study, the deep MLP model with four hidden layers was compared with classical machine learning models such as Support Vector Machines (SVC), Random Forest (RF), Decision Tree (DT), and Logistic Regression (LR). In addition to this, a two-stage ensemble model was proposed. In the first stage of this model, SVC was trained as a classifier to compute prediction probability. In the second stage of the model, these prediction probability scores were used as extra features, and several other machine learning models such as MLP, RF, DT, and LR were trained [17]. One of the aims of the study is to compare traditional models with artificial neural network models. Therefore, traditional machine learning models, which are frequently used in the literature and have achieved successful results in many problems, were chosen in this study. The architecture of the proposed ensemble model is shown in Figure 1.

The first stage of the model aims to compute the prediction probability of all samples in the training dataset. To achieve this goal, 2-fold cross-validation was employed in the initial phase of the proposed model to prevent overfitting. The SVC model was trained using the first fold of the training dataset to compute the prediction probability of the second fold, and conversely, another SVC model was trained using the second fold of the training dataset to compute the prediction probability of the first fold. After calculating the prediction probability of the training dataset, the features were concatenated, and a second model was trained to determine the final class of samples. In the second phase, MLP, RF, DT, and LR models were trained separately. In this study, 10-fold cross-validation was implemented instead of the holdout method; therefore, this process was applied individually for each fold of the dataset [17].

#### 2.3.3. Model Training and Validation

The model was trained using all of the features, employing techniques such as cross-validation to optimize model parameters and prevent overfitting. For this purpose, 10-fold cross-validation was used to assess the performance of our models. A separate hyper-parameter optimization process was applied for each fold using a random search technique. An inner 3-fold cross-validation approach was used while optimizing the hyper-parameters of each fold. For this purpose, the number of neurons in hidden layers, learning rate, and number of epochs in MLP models; maximum depth and criterion in DT models; C and gamma values in SVC models; number of estimators, maximum depth, and minimum samples split in RF models; C value and maximum iteration in LR models were optimized. The scikit-learn library, available in Python, was used for cross-validation, hyperparameter optimization, and the implementation of machine learning models in this study. Detailed information related to the optimum hyperparameters for each fold were given in a Supplementary File (Tables S1 and S2) [18].

#### 2.3.4. Explainability Integration

SHAP (SHapley Additive exPlanations) is a robust interpretability approach inspired by cooperative game theory, particularly intended to break down and interpret the contributions of each feature to a model’s predictions. By computing SHAP values, it gives a clear and consistent approach to understanding the influence of each feature on the final output of the model. This interpretability is especially critical in clinical decision support systems, where knowing the logic behind a model’s prediction may dramatically affect patient treatment. SHAP values have both global and local interpretability, meaning they can explain the overall effect of characteristics across all predictions as well as the impact on individual predictions. This dual viewpoint promotes transparency and confidence in the model, enabling physicians to make educated judgments based on full knowledge of how numerous characteristics, such as patient demographics, test findings, and medical history, contribute to the prediction. Consequently, SHAP values serve a critical role in ensuring that complicated models employed in healthcare settings are both interpretable and dependable, eventually enabling improved clinical results [19].

#### 2.3.5. Model Evaluation

The evaluation of the model was undertaken by performing applicable performance criteria, including accuracy, precision, F1-score, and F-beta scores, to objectively examine its efficacy. These metrics are significant as they provide a full study of the model’s potential to reliably forecast diabetic retinopathy based on clinical data. Specifically, accuracy measures the proportion of total correct predictions made by the model, precision evaluates the correctness of positive predictions, the F1-score represents the harmonic mean of precision and recall, and F-beta scores are a generalization of the F1-score that weight recall more heavily, making them particularly valuable in scenarios in which failing to detect true positives has severe consequences. The beta score was assigned a value of 0.5 for the F-beta score [20].

#### 2.3.6. Interpretation of Results and Clinical Validation

Within the framework of a clinical setting, the SHAP values were evaluated to explain and validate the judgments made by the model. It was determined that the model’s predictions were both medically sound and beneficial for clinical applications by correlating these findings with established clinical recommendations and published research [21].

### 2.4. Data Analysis

Data were analyzed using the statistical software R (version 4.1.2, R Foundation for Statistical Computing, Vienna, Austria). Before statistical testing, data cleaning procedures were performed to ensure accuracy and completeness. Variables with erroneous or non-numeric entries were corrected or excluded based on the context. The normality assumption was tested by the Shapiro–Wilk test. For the quantitative variables, differences across the NDR, NPDR, and severe PDR groups were tested using the Kruskal–Wallis H test, a non-parametric method suitable for data not following a normal distribution. This test was chosen to assess the overall difference among the three groups for each variable of interest. Post-hoc pairwise comparisons were conducted using the Mann–Whitney U test with Bonferroni correction to control for Type I error across multiple tests. The significance level for all analyses was set at α = 0.05, with the Bonferroni adjustment applied based on the number of comparisons. The relationship between categorical variables was examined using the Chi-square test of independence. This test was specifically applied to assess the association between gender and the retinopathy groups. Results are reported with median and interquartile range (IQR) values for continuous variables to provide robust measures of central tendency and variability, given the non-normal distribution of the data. Statistical significance, test statistics, and *p*-values are presented to detail the outcomes of the analyses conducted. For the data analysis and modeling, we used the SHAP, and scikit-learn libraries in Python.

## 3. Results

The study sample consisted of T2DM patients (n = 317), comprising NDR (n = 143) and DR (n = 174) individuals. DR patients were further separated into two groups according to the status of the problems. These included the NPDR (n = 123) and PDR (n = 51) groups. Significant age differences were observed across the groups (H = 23.34, *p* < 0.00001). The median age and interquartile ranges (IQR) for each group were as follows: NDR (median = 55, IQR = [50, 60]), NPDR (median = 58, IQR = [53, 63]), and PDR (median = 60, IQR = [55, 65]). Post-hoc analyses using the Bonferroni correction showed significant differences between NDR and NPDR (*p* = 0.0000126), and NDR and PDR (*p* = 0.000551), but not between NPDR and PDR (*p* = 0.865). Significant differences were found in the HbA1c levels (H = 17.42, *p* < 0.0002). The median HbA1c values were as follows: NDR (6.0%, IQR = [5.7, 6.3]), NPDR (7.2%, IQR = [6.8, 7.6]), and PDR (7.5%, IQR = [7.1, 8.0]). Pairwise comparisons indicated significant differences between NDR and NPDR (*p* = 0.000122) and between NDR and PDR (*p* = 0.00418), but not between NPDR and PDR (*p* = 0.824). Glucose levels differed across the groups (H = 10.01, *p* = 0.0067). Median glucose levels were as follows: NDR (90 mg/dL, IQR = [85, 95]), NPDR (120 mg/dL, IQR = [110, 130]), and PDR (125 mg/dL, IQR = [115, 135]). The Bonferroni adjusted pairwise tests revealed significant differences between NDR and NPDR (*p* = 0.00155), but not between NDR and PDR (*p* = 0.12) or NPDR and PDR (*p* = 0.617). Creatinine levels also showed significant differences (H = 27.06, *p* < 0.000001). The median values were as follows: NDR (0.9 mg/dL, IQR = [0.8, 1.0]), NPDR (1.1 mg/dL, IQR = [1.0, 1.2]), and PDR (1.3 mg/dL, IQR = [1.2, 1.4]). Significant differences were found between all paired groups: NDR and NPDR (*p* = 0.00816), NDR and PDR (*p* = 0.000000692), and NPDR and PDR (*p* = 0.000874). We examined the association between gender and the retinopathy groups using a Chi-square test, which indicated no significant association (χ^2^ = 0.768, *p* = 0.681).

Table 1 indicates the analysis results of the non-hybrid models. In addition to this, two-stage ensemble models were also trained in this study. As explained before, in the first stage of this model, SVC was trained as a classifier to compute prediction probability. In the second stage, several other models were trained using these prediction probability scores as an extra feature. As a result of this process, four models, SVC + RF, SVC + DT, SVC + LR, and SVC + MLP, were trained.

Table 2 shows the performance metrics of the ensemble models trained using the T2DM dataset. According to the results in this table, there is an increase in performance measures when applying the proposed two-layer ensemble approach to any ML model used in the study. In addition, the deep neural network model achieved more successful results both in single models and hybrid models. In this regard, it is predicted that utilizing the deep MLP model would be most suitable for designing a biomarker. In designing this biomarker, the models are elucidated using the SHAP method to assess the impact of each feature utilized in the machine learning model on the success rate. The impact of features computed by SHAP using the MLP model, which outperformed the other models, is shown in the following figures.

**Figure 2:**
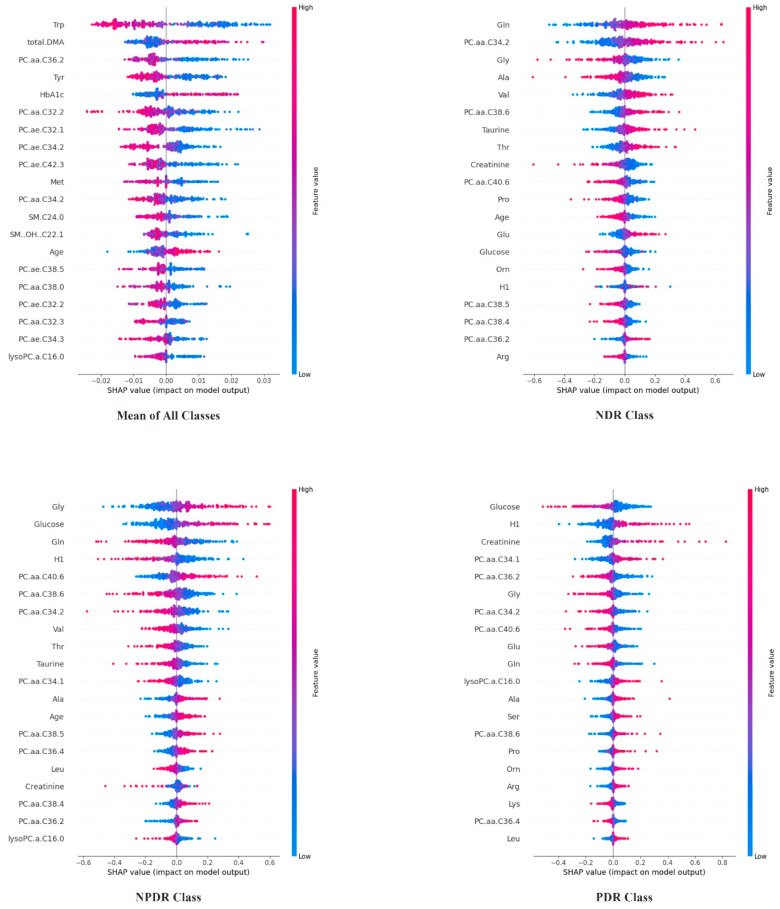
SHAP (SHapley Additive exPlanations) importance values for the solo MLP models, showing the overall feature importance and the importance for each class of DR. The features include glucose, glycine, and age, which show significant importance across all classes (NDR, NPDR, PDR). This analysis highlights the key metabolic and physiological markers influencing the model’s predictions, emphasizing the critical role of these biomarkers in DR pathology.

Figure 2 illustrates the different patterns of feature importance across the DR classes, indicating that certain biochemical and physiological parameters are more relevant for certain conditions. Glucose and glycine show significant importance in all classes but are particularly impactful in the NPDR class. The analysis of SHAP values across different DR classes provides insights into the model’s behavior and decision-making process. The model leverages a complex interaction of features where metabolic and age-related factors like glucose, glycine, and age appear consistently across the classes, suggesting their universal importance in the pathology of DR. The prominence of specific metabolites and amino acids such as taurine, creatinine, and various phosphatidylcholines highlights the potential metabolic underpinnings of DR progression. This could suggest pathways for targeted therapeutic interventions or for biomarkers in clinical settings. In the PDR class, a marked influence of creatinine and the phosphatidylcholine molecules suggest a shift towards more systemic and nephrological influences as DR progresses to more severe forms. This shift is crucial for understanding how DR could be connected to broader systemic conditions.

**Figure 3:**
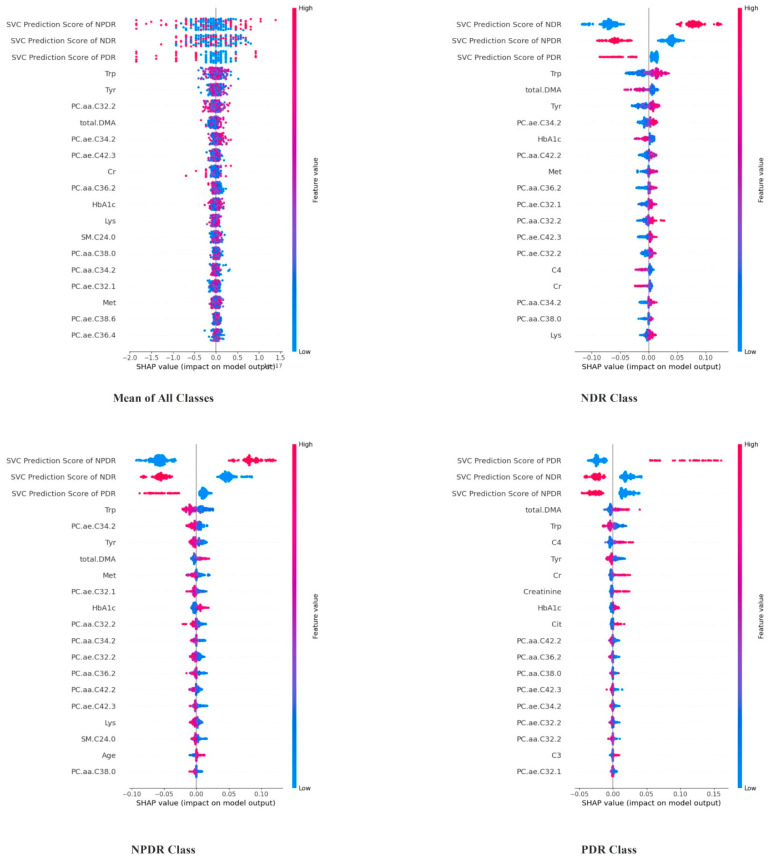
SHAP importance values for the hybrid MLP models, depicting the influence of various biochemical and physiological features across different DR classes. Features such as HbA1c, tyrosine (Tyr), and phosphatidylcholine molecules (e.g., PC.ae.C36.2) appear frequently, indicating their significant roles in the model’s decision-making process. This figure shows how different features carry varying weights depending on the DR severity, with certain markers being particularly relevant for identifying more severe stages of DR.

Figure 3 demonstrates which biochemical and physiological features are most influential across different DR classes. Features such as HbA1c, Tyr (Tyrosine), and various phosphatidylcholine molecules (e.g., PC.ae.C36.2) appear frequently across the plots, indicating their significant role in the model’s decision-making process. Certain features have more pronounced impacts in specific DR classes. HbA1c and Tyr have a more substantial influence in the PDR class compared to the NDR and NPDR classes. This suggests that these features may be particularly relevant for identifying more severe stages of diabetic retinopathy. The comparison between classes highlights that different features carry different weights depending on the severity of the condition. Creatinine and citrate show significant importance in the NDR class, which might indicate their utility in distinguishing no retinopathy from some degree of retinopathy. It may be determined from the study findings that the two-stage hybrid strategy delivers more favorable outcomes. Upon inspection of feature significance in the hybrid model, it becomes obvious that the probabilities produced from the machine learning model applied in the second stage exert the most significant effect on the final class prediction. Considering these data, it is inferred that the boost in the performance of the two-stage hybrid approach may be attributable to the prediction probabilities provided by the first-stage method.

## 4. Discussion

The present study presents a comprehensive review of solo and hybrid machine learning models in DR prediction and analysis using the T2DM dataset. A substantial boost in prediction performance measures was obtained with the deployment of two-stage ensemble models over non-hybrid, solo models. This suggests a strategic benefit of combining various learning algorithms to increase prediction accuracy, precision, and other performance measurements.

The solo models analyzed (SVC, RF, DT, LR, and MLP) displayed commendable individual performances, with the MLP model outperforming others in terms of accuracy, precision, and F-scores. The MLP’s superior performance aligns with findings from a scientific article, which reported that deep learning models often outstrip traditional machine learning models in medical image analysis due to their ability to learn complex patterns from large datasets [22]. One of the main purposes of the ensemble method is to correct errors made by one method using another method. In this study, the prediction probabilities calculated by the MLP were used as input for a second model to create an ensemble method. When examining studies in the literature, it has been observed that ensemble methods generally achieve more successful results than individual methods. Another factor that affects the performance of the model is the individual success of each method used in the ensemble. Therefore, it was expected that the best score observed in our study would be obtained with an ensemble method.

When examining the ensemble models (SVC + RF, SVC + DT, SVC + LR, and SVC + MLP), the SVC + MLP configuration showed the highest improvement in all metrics. This enhancement is consistent with that reported in other research, which found that layering different types of models could lead to more robust predictions in biomedical applications by capturing diverse patterns that solo models might miss [23].

Furthermore, using the SHAP method to interpret model predictions provided insightful revelations about the feature importance in disease progression, particularly in different classes of DR. The significant roles of glucose, glycine, and age across all DR classes suggest their universal importance in DR pathology. This finding is corroborated by research that highlighted metabolic and age-related factors as critical in DR progression [24]. Moreover, the analysis revealed varying impacts of specific biochemical markers across different DR severity levels. Creatinine and various phosphatidylcholine molecules exhibited higher importance in more severe DR classes (PDR), similar to observations by a medical study, which suggested a link between nephrological markers and severe DR conditions [25].

The results of this study demonstrate considerable variation in age, HbA1c, glucose, and creatinine levels throughout different phases of diabetic retinopathy, underscoring the relevance of these biomarkers in monitoring disease progression. Notably, the increase in median HbA1c, glucose, and creatinine values from non-proliferative to severe proliferative diabetic retinopathy suggests a link with the deterioration of the disease condition. These findings are consistent with recent research suggesting that prolonged exposure to high glucose levels could improve the severity of retinopathy, presumably due to increased oxidative stress and vascular damage inside the retina [26]. Moreover, the large age differences identified across the groups further support the hypothesis that the risk and development of diabetic retinopathy worsen with age. This is under the larger awareness within the profession that older age is a significant risk factor for the development of more severe diabetes-related problems [27]. Our study identified no significant gender differences in the course of diabetic retinopathy, suggesting that the physiologic consequences of diabetes on retinal health could be similar across genders. This accords with clinical research that has questioned established ideas about gender discrepancies in diabetes outcomes, claiming instead that lifestyle and medication adherence may play more major roles [28]. These findings are essential for doctors and academics alike, as they provide knowledge that can change screening and monitoring protocols for diabetic retinopathy. By recognizing the importance of age, HbA1c, glucose, and creatinine as indicators, healthcare practitioners can optimize patient outcomes through earlier intervention and personalized treatment programs [29]. In contrast, some studies have reported minimal improvements in model performance when combining classifiers in the same way. This discrepancy could be attributed to differences in datasets, feature sets, or model tuning, highlighting the context-dependent nature of machine learning applications in healthcare [30,31].

Glucose consistently emerges as the most important metabolite in both solo and hybrid MLP models in all stages of DR. This finding shows that the role of diabetes mellitus and high consumption in DR development is well-established and consistent. Elevated glucose levels are a hallmark of diabetes and are directly linked to nerve damage and subsequent retinal problems. Chronic hyperglycemia leads to the production of advanced glycation end products (AGEs), which accumulate and cause structural and functional abnormalities in retinal blood vessels and this process leads to increased vascular permeability, microaneurysms growth, and end completely the blood–retinal barrier collapses. In addition, excess glucose levels can activate multiple signaling pathways that exacerbate inflammation and oxidative stress, exacerbating retinal damage. The consistent role of glucose as a key metabolic factor in DR models in its many phases emphasizes the critical importance of strict glycemic control in terms of control and prevention development of this disorder. In order to reduce the risk of DR progression and consequences, the associated metabolism may, ultimately, improve patient outcomes and quality of life. This insight into the primary role of glucose also highlights the importance of continued research and innovation to build effective ways to maintain adequate glycemic emphasize levels in diabetics [32]. Managing glucose levels is thus paramount in preventing DR progression. Glycine is another significant metabolite, particularly highlighted in the Hybrid MLP models. Glycine’s role in neurotransmission and as a metabolic regulator suggests its involvement in the metabolic disturbances associated with diabetes [33]. Elevated glycine levels may indicate an impaired glucose metabolism and increased oxidative stress, contributing to DR development. HbA1c, a measure of long-term glycemic control, is crucial in predicting DR stages. Its importance reflects the necessity for sustained glucose management to mitigate DR risks. Elevated HbA1c levels are strongly correlated with the severity of retinopathy [34]. Phosphatidylcholines, including PC.aa.C34.2 and PC.aa.C38.6, are particularly prominent in the Solo MLP models for the NPDR and PDR classes. These metabolites are essential components of cell membranes and play significant roles in lipid metabolism. Altered PC levels suggest disruptions in lipid homeostasis and cellular integrity, contributing to retinal damage [35]. Several amino acids, such as glutamine (Gln), alanine (Ala), valine (Val), threonine (Thr), and arginine (Arg), are also highlighted based on the comprehensive analysis. These amino acids are vital for protein synthesis and energy metabolism, and their altered levels can indicate broader metabolic dysregulation in diabetes. Creatinine, a marker of renal function, is significant in both the NPDR and PDR classes. This is consistent with the high prevalence of diabetic nephropathy in advanced DR stages. Age also emerges as an important factor, reflecting the increased risk of DR with advancing age [36,37]. Other metabolites, such as ornithine and proline, which are involved in the urea cycle and amino acid metabolism, as well as Trp and Tyr, precursors to neurotransmitters, suggest potential links between metabolic and neurodegenerative processes in diabetes [38].

The study’s findings emphasize the promise of advanced machine learning methodologies, particularly hybrid models, in enhancing the projected accuracy and interpretability of health-related outcomes. Ensemble models and deep learning, along with interpretative techniques like SHAP, can greatly contribute to understanding intricate illness causes and improving diagnostic processes in clinical settings. Future studies might examine the integration of other, different models and the assessment of their interpretability to further enhance the predictions and insights produced by such sophisticated analytical tools [39].

The superior performance of hybrid models, particularly the SVC + MLP model, suggests that these models could significantly enhance DR’s diagnostic accuracy. This precision is critical in distinguishing between the various stages of DR, allowing for earlier and more precise interventions, and potentially reducing the progression to more severe stages that require invasive treatments. The integration of these models into CDSS can provide ophthalmologists with powerful tools to analyze retinal images more efficiently. This integration can aid in making quicker and more accurate decisions, particularly in areas with limited access to specialized healthcare providers [40]. The ability of the models to identify early signs of DR and to elucidate the metabolic and physiological markers associated with its progression offers a pathway to preventive healthcare strategies. By identifying at-risk individuals early, preventative measures can be taken sooner, which may include lifestyle and dietary changes, as well as closer monitoring of glucose and blood pressure levels. While the current study demonstrates significant potential, the scalability and adaptability of these models in different clinical settings remain to be tested. The models need to be validated not only across various demographics but also across different equipment and settings to ensure they maintain accuracy without high-grade, specialized equipment. Further research could explore combining the predictive power of machine learning models with other modalities like genetic testing or biomarker analysis to enhance predictive accuracy further. Additionally, longitudinal studies could assess how interventions based on model predictions affect patient outcomes over time. The hybrid SVC + MLP model outperformed the solo models in predicting and assessing DR, which may be attributed to various theoretical benefits inherent to ensemble learning. Ensemble learning integrates various learning algorithms to produce higher prediction performance than could be achieved from any of the component models alone. This strategy harnesses the strengths and mitigates the limitations of individual models, resulting in more robust and accurate forecasts.

The uncovered indicators, including glucose, glycine, HbA1c, and creatinine, might possibly be incorporated into existing clinical practice to promote early identification and tailored treatment methods for DR. For practical implementation, these biomarkers need to be verified in larger, independent cohorts to ensure their reliability and generalizability. This validation method incorporates longitudinal studies to evaluate the evolution of DR in varied groups and contexts. Additionally, incorporating these biomarkers into clinical decision support systems (CDSS) may benefit ophthalmologists in making more accurate and timely judgments, especially in resource-limited situations. The research implies that early identification of at-risk patients using these indicators might lead to preventive actions, such as lifestyle adjustments and tight glucose control, eventually improving patient outcomes. Therefore, future research should concentrate on confirming these biomarkers, understanding their significance in DR pathophysiology, and creating rigorous guidelines for their adoption in normal clinical practice. This will ensure that the encouraging findings from machine learning models translate into actual gains in controlling and preventing DR in clinical settings.

Recent research primarily concentrates on the growing role of hypertension and environmental variables in DR development, as well as the newest breakthroughs in AI applications in metabolomics. Notably, the inclusion of new studies addressing the relationship between metabolic diseases and hypertensive situations should provide a more comprehensive understanding of DR pathogenesis. Additionally, there is a need to mention significant references describing comparable AI applications in metabolomics, which would frame this work within the larger context of contemporary technical breakthroughs. Integrating these updates will boost the manuscript’s relevancy and highlight its alignment with cutting-edge research on this quickly evolving subject [41,42].

The clinical efficacy of the machine learning models addressed in this work, particularly the hybrid models such as SVC + MLP, indicates a promising improvement in the early diagnosis and management of DR. The findings demonstrate that these models not only boost diagnostic accuracy but also precision, enabling earlier intervention options that are critical in limiting the progression of DR [43]. This is particularly advantageous for places where access to expert healthcare practitioners is restricted. Furthermore, implementing these models in real-world clinical settings could significantly streamline the screening process, making it faster and more reliable. This would allow for a broader, more effective deployment of resources, potentially reducing the overall healthcare burden associated with late-stage DR treatments. By integrating these advanced predictive models into existing clinical workflows, there is an opportunity to transform current DR management practices, emphasizing preventative care and personalized treatment plans based on precise, data-driven insights. However, to realize their full potential, these models must undergo extensive clinical validation to ensure their efficacy and reliability across diverse patient demographics and varying clinical environments.

## 5. Conclusions

The use of XAI in DR utilizing the implemented models indicates promising outcomes in enhancing the accuracy and efficiency of diagnosis and therapy. Through the construction of interpretable algorithms, healthcare personnel may obtain greater insights into the decision-making process of AI systems, leading to increased confidence and adoption of these technologies in clinical practice. By offering open explanations for the predictions and suggestions made by AI, clinicians may better comprehend the underlying rationale and perhaps unearth a new understanding about the condition. Overall, the incorporation of explainable AI in DR not only boosts diagnostic performance but also promotes collaboration between human specialists and ML systems, eventually enhancing our knowledge and management of this sight-threatening disorder.

## Figures and Tables

**Table 1:** Performance metric findings of Solo models.

Model	Accuracy (%)	Precision (%)	F1-Score (%)	F-Beta Score (%)
SVC	84.54	80.81	81.68	81.06
RF	85.48	83.05	83.88	83.37
DT	85.17	83.20	84.15	83.54
LR	83.59	80.39	81.41	80.70
MLP	86.75	84.80	85.48	85.06

**Table 2:** Performance metric findings of hybrid models.

Model	Accuracy (%)	Precision (%)	F1-Score (%)	F-Beta Score (%)
SVC + RF	86.11	83.39	84.38	83.64
SVC + DT	85.80	83.48	84.75	83.89
SVC + LR	83.91	80.79	81.41	80.94
SVC + MLP	89.58	87.18	88.20	87.55

## Data Availability

Data can be requested from the corresponding author upon appropriate request.

## Supplementary Materials

The following supporting information can be downloaded at: https://www.mdpi.com/article/10.3390/diagnostics14131364/s1, Table S1: Optimum hyper-parameter details of the solo models; Table S2: Optimum hyper-parameter details of the hybrid models.
