# Supplementary material for: Hybrid Explainable Artificial Intelligence Models for Targeted Metabolomics Analysis of Diabetic Retinopathy
Source: Diagnostics (Basel). 2024 Jun 27;14(13):1364. doi: 10.3390/diagnostics14131364 (PMC11241009; doi:10.3390/diagnostics14131364)
Supplement: Supplementary file 1 [file diagnostics-14-01364-s001.zip › diagnostics-3044716-supplementary.pdf]

Model Name	Hyper-Parameter Name	Optimum Value for Fold 1	Optimum Value for Fold 2	Optimum Value for Fold 3	Optimum Value for Fold 4	Optimum Value for Fold 5	Optimum Value for Fold 6	Optimum Value for Fold 7	Optimum Value for Fold 8	Optimum Value for Fold 9	Optimum Value for Fold 10
SVC	C	128	0.5	8	0.001953125	0.03125	0.125	9	0.015625	0.3125	0.25
	gamma	16	0.001953125	256	128	0.125	0.00390625	0.125	512	0.015625	1
RF	estimators	300	350	200	300	100	350	150	300	250	150
	max_depth	20	2	15	5	10	5	20	2	10	20
LR	C	0.03125	0.015625	0.25	0.0009765625	64	0.03125	32	32	0.5	0.0625
	Max iteration	737	821	207	918	653	539	748	230	913	84
DT	max_depth	7	5	10	9	15	4	13	1	7	15
	criterion	entropy	Log_loss	gini	entropy	Log_loss	gini	Log_loss	Entropy	Log_loss	Gini
MLP	# neuron	92, 83, 240, 144	174, 159, 201, 142	269, 97, 289, 149	102, 167, 239, 287	153, 193, 98, 210	237, 245, 198, 153	94, 99, 70, 245	77, 92, 217, 221	97, 265, 109, 284	109, 206, 234, 79
	learing rate	0.005	0.05	0.03	0.0001	0.01	0.0001	0.01	0.001	0.05	0.005
	epoch	106	291	136	207	144	214	291	252	285	264

Table S1. Optimum hyper-parameter Details of the solo models

Model Name	Hyper-Parameter Name	Optimum Value for Fold 1	Optimum Value for Fold 2	Optimum Value for Fold 3	Optimum Value for Fold 4	Optimum Value for Fold 5	Optimum Value for Fold 6	Optimum Value for Fold 7	Optimum Value for Fold 8	Optimum Value for Fold 9	Optimum Value for Fold 10
SVC+RF	estimators	250	150	350	50	350	100	250	250	250	150
	max_depth	5	10	2	5	5	15	15	10	10	20
SVC+LR	C	0.0625	0.125	0.25	0.5	0.0625	0.125	8	0.001953125	0.125	0.001953125
	Max iteration	499	764	918	727	777	811	455	228	785	380
SVC+DT	max_depth	7	9	8	15	7	12	1	9	7	9
	criterion	Log_loss	Entropy	Log_loss	Entropy	Log_loss	Entropy	Gini	Entropy	Gini	Log_loss
SVC+MLP	# neuron	217, 288, 174, 259	245, 230, 200, 179	285, 147, 295, 268	230, 152, 175, 169	250, 243, 208, 158	178, 196, 115, 225	103, 209, 174, 158	75, 108, 75, 266	115, 76, 147, 228	241, 94, 275, 296
	learing rate	0.05	0.005	0.05	0.01	0.01	0.001	0.0003	0.0001	0.005	0.0005
	epoch	126	202	201	287	206	198	174	288	116	160

Table S2. Optimum hyper-parameter Details of the hybrid models
